# Ruminant and chicken: important sources of campylobacteriosis in France despite a variation of source attribution in 2009 and 2015

**DOI:** 10.1038/s41598-018-27558-z

**Published:** 2018-06-18

**Authors:** Amandine Thépault, Valérie Rose, Ségolène Quesne, Typhaine Poezevara, Véronique Béven, Edouard Hirchaud, Fabrice Touzain, Pierrick Lucas, Guillaume Méric, Leonardos Mageiros, Samuel K. Sheppard, Marianne Chemaly, Katell Rivoal

**Affiliations:** 1 Hygiene and Quality of Poultry & Pork Products Unit, Laboratory of Ploufragan-Plouzané, French Agency for Food Environmental and Occupational Health & Safety (Anses), Ploufragan, France; 2 University of Rennes 1, Rennes, France; 3 Viral Genetics & Biosafety Unit, Laboratory of Ploufragan-Plouzané, French Agency for Food Environmental and Occupational Health & Safety (Anses), Ploufragan, France; 4 The Milner Centre for Evolution, Department of Biology and Biochemistry, University of Bath, Claverton Down, Bath, United Kingdom; 5 Swansea University, Medical School, Institute of Life Science, Singleton Campus, Swansea, United Kingdom; 6 Department of Zoology, University of Oxford, South Parks Road, Oxford, OX1 3PS United Kingdom

## Abstract

Pathogen source attribution studies are a useful tool for identifying reservoirs of human infection. Based on Multilocus Sequence Typing (MLST) data, such studies have identified chicken as a major source of *C*. *jejuni* human infection. The use of whole genome sequence-based typing methods offers potential to improve the precision of attribution beyond that which is possible from 7 MLST loci. Using published data and 156 novel *C*. *jejuni* genomes sequenced in this study, we performed probabilistic host source attribution of clinical *C*. *jejuni* isolates from France using three types of genotype data: comparative genomic fingerprints; MLST genes; 15 host segregating genes previously identified by whole genome sequencing. Consistent with previous studies, chicken was an important source of campylobacteriosis in France (31–63% of clinical isolates assigned). There was also evidence that ruminants are a source (22–55% of clinical isolates assigned), suggesting that further investigation of potential transmission routes from ruminants to human would be useful. Additionally, we found evidence of environmental and pet sources. However, the relative importance as sources varied according to the year of isolation and the genotyping technique used. Annual variations in attribution emphasize the dynamic nature of zoonotic transmission and the need to perform source attribution regularly.

## Introduction

*Campylobacter* spp. are regarded as the most common foodborne bacterial zoonosis in Europe^1^, despite potential underestimation due to underreporting of cases^2^. In France, *C*. *jejuni* is responsible for nearly 80% of human infections while *C*. *coli* accounts for around 15%^3^. The economic burden of campylobacteriosis has been estimated to 2.4 billion euros annually in Europe^4^, with estimates of £50 million in 2008–2009 in the United Kingdom^5^ and 82 million euros in the Netherlands in 2011^6^.

*Campylobacter* spp. are frequent colonizers of the digestive tract of domesticated animals such as livestock^7–10^ and pets^11,12^, as well as wild birds^13–15^, and have been isolated from environmental waters sources^16,17^. Accurately quantifying the relative importance of each *Campylobacter* reservoir in human infections constitutes an important aim in public health to develop control strategies to decrease the human and economic burden of campylobacteriosis. Previous source attribution studies, principally based upon Multilocus sequence typing (MLST) data^18^ which consists in the sequencing and the allele designation of 7 housekeeping genes of *C*. *jejuni*, have identified chicken as a major source of human infection worldwide, while ruminants, pets and environmental sources are also implicated^19–22^. However, MLST-based attribution has limited efficacy for source attribution of clinical cases from clonal complexes and sequence type that are isolated from multiple hosts, since they show identical allelic variations in the 7 studied genes^23^. Recently, a pan genome approach was used to investigate host signal within 411 *C*. *jejuni* genomes, and 15 markers were identified as promising candidates for source attribution as they allowed the segregation of *C*. *jejuni* isolates according to their host^24^. In addition, comparative genomic fingerprinting approach (CGF) has also been developed to genotype *C*. *jejuni* isolates with a high resolution^25^ and has been extensively used in Canada for routine surveillance of campylobacteriosis^25–27^. The CGF40 approach, which consists in the assessment of the presence/absence of 40 genes belonging to the accessory genome of *C*. *jejuni* through gene amplification, showed concordant results with MLST with a higher discriminatory power^28^, and could be an interesting alternative to MLST by potentially improving the accuracy of source attribution studies.

Here, we assessed the accuracy of attributions of *C*. *jejuni* isolates to their source based on MLST, CGF40 profiles and the 15 host segregating markers, and used the most accurate methods to identify the most likely origin of French campylobacteriosis from 2009 and 2015. Isolates originating from chicken, ruminant, pets, environmental waters and wild birds were considered as potential sources of human infection in the analysis.

## Results

### Clinical, animal, and environmental isolates genotyping using CGF40, MLST and whole genome sequencing (WGS)

*C*. *jejuni* clinical isolates from 2009 appeared to be highly diverse with 85 CGF40 clusters based on 100% of similarity between isolates, and 62 STs^29^. Clinical isolates from 2015 were also highly diverse with 229 CGF40 genotypes found. In addition, MLST performed on a subset of these clinical isolates (n = 79) using WGS, revealed 54 different STs, and 79% of the clinical isolates belonged to the 12 most common clonal complexes found (ST-21, ST-206, ST-257, ST-353, ST-48, ST-464, ST-22, ST-283, ST-42, ST-45, ST-52 and ST-658 complexes).

A total of 1,618 animal and environmental *C*. *jejuni* isolates from putative sources of human infection (i.e. chicken, ruminant, environment, and pets) constituted the comparison data set of CGF40 genotypes, while the comparison data sets of MLST and host-segregating markers profiles comprised respectively 857 and 740 isolates characterized in previous studies (Supplementary Table S1).

### Accuracy of the several genotypes data in source attribution through self-attribution tests

**Figure 1:**
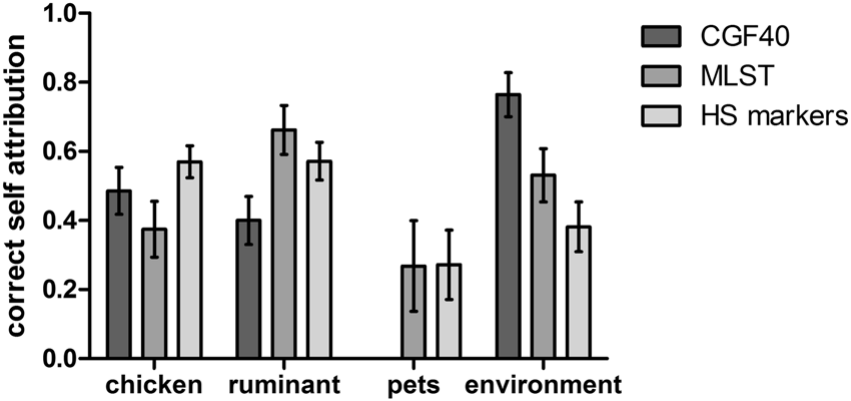
Correct self-attribution rates of *C*. *jejuni* isolates from 4 putative contamination sources based on genomic data obtained with CGF40, MLST or WGS (15 host segregating markers).

The accuracy of the different genotyping methods in source attribution was assessed with isolates of a known origin. Self-attributions were performed on randomly selected subsets of isolates from each of the 4 putative contamination sources and the rates of correct self-attributions are shown in the Fig. 1. The probabilities of assignment of these isolates to the others sources are presented in the Table 1, as well as their confidence interval at 95%. The probabilities of correct self-attribution using CGF40 markers for source attribution were estimated to 49% in chicken (CI95% = 0.418, 0.553), 40% in ruminant (CI95% = 0.330, 0.469), 76% in environment (CI95% = 0.701, 0.828), and 0% (CI95% = 0.00, 0.00) in pets isolates. However, MLST allowed significantly higher correct self-attribution rates than CGF40 within ruminant (66%; CI95% = 0.591, 0.733) and pets isolates (27%; CI95% = 0.137, 0.399). Nevertheless, MLST showed a significantly lower rate within environmental isolates (53%; CI95% = 0.454, 0.608) than CGF40 since there was no overlap between their confidence interval at 95%, while a similar rate was observed within chicken isolates with 37% (CI95% = 0.294, 0.455) of correct self-attribution. Finally, the use of the 15 host-segregating markers in source attribution gave a correct self-attribution of 57% (CI95% = 0.524, 0.616) in chicken, which is significantly higher than using MSLT, while correct self-attribution rates, similar to MLST, were observed in ruminant (57%; CI95% = 0.517, 0.626), environmental (38%; CI95% = 0.309, 0.453) and pets isolates (27%; CI95% = 0.172, 0.372).

**Table 1:** Self-attribution of *C*. *jejuni* isolates from 4 putative sources of human infections using molecular data from CGF40, MLST or WGS using 15 host-segregating markers (HS markers).

	CGF40				MLST				WGS (15 HS markers)			
	Chicken	Ruminant	Environment	Pets	Chicken	Ruminant	Environment	Pets	Chicken	Ruminant	Environment	Pets
Chicken	**0**.**49** (0.42–0.55)	0.23 (0.17–0.29)	0.27 (0.21–0.34)	0.01 (−0.01–0.03)	**0**.**37** (0.29–0.46)	0.27 (0.19–0.34)	0.18 (0.10–0.26)	0.18 (0.12–0.24)	**0**.**57** (0.52–0.62)	0.11 (0.06–0.16)	0.12 (0.03–0.21)	0.20 (0.11–0.30)
Ruminant	0.19 (0.10–0.28)	**0**.**40** (0.33–0.47)	0.14 (0.06–0.23)	0.26 (0.19–0.34)	0.11 (0.05–0.17)	**0**.**66** (0.59–0.73)	0.08 (0.03–0.13)	0.15 (0.06–0.23)	0.28 (0.21–0.34)	**0**.**57** (0.52–0.63)	0.06 (0.02–0.11)	0.09 (0.04–0.14)
Environment	0.24 (0.17–0.30)	0.00 (0.00–0.00)	**0**.**76** (0.70–0.83)	0.00 (0.00–0.00)	0.08 (0.04–0.13)	0.01 (0.00–0.01)	**0**.**53** (0.45–0.61)	0.38 (0.30–0.45)	0.20 (0.07–0.33)	0.05 (0.02–0.07)	**0**.**38** (0.31–0.45)	0.37 (0.28–0.47)
Pets	0.38 (0.32–0.45)	0.05 (0.03–0.08)	0.56 (0.49–0.63)	**0**.**0** (0.0–0.0)	0.33 (0.28–0.37)	0.25 (0.22–0.27)	0.16 (0.05–0.27)	**0**.**27** (0.14–0.40)	0.32 (0.22–0.41)	0.11 (0.07–0.17)	0.30 (0.22–0.38)	**0**.**27** (0.17–0.37)

Host populations in bold letters are populations for which isoaltes were tested in self-attribution tests. Self-attribution probabilities for a same host population are presented in line.

### Source attribution of *C. jejuni* clinical isolates from 2009 and 2015

**Figure 2:**
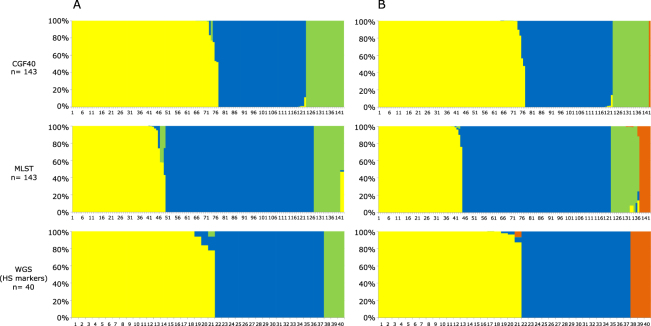
Estimated source probabilities of French clinical isolates from 2009 using three genotyping methods for source attribution. (**A**) Probabilities of clinical isolates to originate from 3 putative sources (yellow: chicken; blue: ruminant, and green: environment), (**B**) Probabilities of clinical isolates to originate from 4 putative sources (yellow: chicken; blue: ruminant, green: environment, orange: pets). Each vertical bar represents one isolate, and the color of the bar shows the estimated probability that this isolate originates from each of the potential sources.

**Figure 3:**
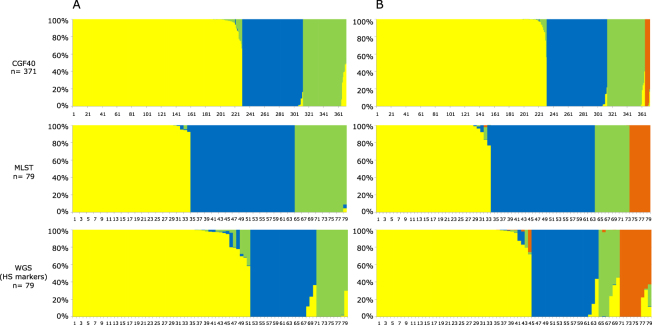
Estimated source probabilities of French clinical isolates from 2015 using three genotyping methods for source attribution. (**A**) Probabilities of clinical isolates to originate from 3 putative sources (yellow: chicken; blue: ruminant, and green: environment), (**B**) Probabilities of clinical isolates to originate from 4 putative sources (yellow: chicken; blue: ruminant, green: environment, orange: pets). Each vertical bar represents one isolate, and the color of the bar shows the estimated probability that this isolate originates from each of the potential sources.

The probabilistic assignments of each clinical case from 2009 and 2015 to the different putative contamination sources were calculated using STRUCTURE software and are shown in Figs 2 and 3. Regarding clinical isolates from 2009 (Fig. 2A), MLST attributed 55% (CI95% = 0.468, 0.632) of isolates to ruminant, 34% (CI95% = 0.260, 0.413) to chicken and 11% (CI95% = 0.062, 0.163) to the environment. Based on the 15 host-segregating markers, we observed an equivalent attribution of clinical isolates in 2009 to chicken and ruminant with respectively 51% (CI95% = 0.355, 0.673) and 41% (CI95% = 0.253, 0.566), while the implication of the environment was estimated to 8% (CI95% = 0.0, 0.162). Finally, using the CGF40 data to perform source attribution, a higher implication of the chicken reservoir was observed (53%; CI95% = 0.447, 0.609) in clinical cases from 2009, while ruminant and the environment showed respectively 33% (CI95% = 0.253, 0.407) and 14% (CI95% = 0.084, 0.199) of attribution.

When pets were added as a putative source of human contamination in 2009 (Fig. 2B), some clinical isolates were attributed to this source but the global trends remained similar using CGF40 (Chicken: 53% [CI95% = 0.449, 0.613]; Ruminant: 33% [CI95% = 0.252, 0.406]; Environment: 13% [CI95% = 0.077, 0.189]; Pets: 1% [CI95% = 0.0, 0.021]) and MLST (Chicken: 31% [CI95% = 0.231, 0.383]; Ruminant: 55% [CI95% = 0.467, 0.631]; Environment: 10% [CI95% = 0.052, 0.149]; Pets: 4% [CI95% = 0.010, 0.076]). However, when the host segregating markers were used, all previously environment-assigned clinical isolates were attributed to pets while the attributions to chicken and ruminant were equivalent than previously (Chicken: 52% [CI95% = 0.360, 0.681]; Ruminant: 40% [0.245, 0.561]; Environment: 0% [CI95% = 0.00, 0.00]; Pets: 8% [CI95% = 0.00, 0.162]).

Clinical cases from 2015 were then probabilistically assigned to sources (Fig. 3). In MLST-based assignments, 43% (CI95% = 0.318, 0.539) of isolates were attributed to chicken, 38% (CI95% = 0.273, 0.491) to ruminants and 19% (CI95% = 0.101, 0.277) to the environment (Fig. 3A). The 15 host segregating markers allowed the assignment of 63% (CI95% = 0.532, 0.737), 24% (CI95% = 0.146, 0.331) and 13% (CI95% = 0.057, 0.197) of clinical isolates to the chicken, ruminant and environmental reservoirs respectively. These attributions were consistent with the proportions of clinical cases attributed to the chickens (62%; CI95% = 0.573, 0.670), ruminants (22%, CI95% = 0.178, 0.262) and environmental samples (16%, CI95% = 0.122, 0.194) using CGF40 data for source attribution.

When pets were included in the source attribution study as a potential source of human contamination in 2015 (Fig. 3B), global trends were unchanged except for the assignment based on host segregating markers, where 12% (CI95% = 0.050, 0.188) of clinical cases were assigned to pets, while assignment to the environment decreased to 7% (CI95% = 0.018, 0.119). Using CGF40 data or MLST in the STRUCTURE model, 2% (CI95% = 0.005, 0.032) and 8% (CI95% = 0.017, 0.136) of human isolates were respectively assigned to the pets reservoir.

## Discussion

In this study, we attribute the source of clinical *C*. *jejuni* isolates using MLST, CGF40 genotypes and allelic variation within 15 host-segregating markers derived from WGS. While MLST has previously been widely used to assign a source to clinical isolates of *Campylobacter* spp.^19,20,22,30–34^, the use of CGF40 and WGS host-segregating markers are relatively recent^24,35^. The accuracy of each genotyping method was assessed by performing self-attribution tests. In these tests, host-segregating markers allowed the greatest rate of correct assignment of isolates from all hosts to their origin apart from environment isolates for which CGF40 gave a higher probability. These results are not surprising since host-segregating markers were picked for their potential to improve source attribution as they showed the highest rates of correct self-attribution in chicken and ruminant^24^. MLST gave equivalent probabilities of correct-assignment to host-segregating marker analysis in all hosts except for chicken isolates where attribution was lower than the host-segregating markers. Using CGF40, the probability of correct-assignment in chicken isolates was equivalent to the probability using the host segregating markers, but lower probabilities were observed in ruminant and pets isolates.

The difference of accuracy in self-attribution tests according to the genotyping method used, which may trigger differences in source attribution of clinical cases, could be explained by the resolution of data provided by each genotyping method. MLST and host segregating markers provide highly discriminatory data since they assess the allelic variation within each tested gene. For example, there were 35 to 59 different alleles among each MLST genes and from 27 to 169 different alleles in each host-segregating markers within the isolates from this study, while CGF40 produces only binary data (0 or 1) informing on presence or absence of 40 assay genes. Furthermore, data resolution is important especially in a probabilistic model like STRUCTURE which assumes that each host population is characterized by its own set of allelic frequencies, and in which low numbers of markers showing high levels of allelic diversity are more informative than randomly selected markers^36^. Indeed, if the genetic information provided by the genotyping method used to characterize isolates is not sufficient to discriminate isolates from several sources, misattributions of clinical cases to their source can occur using STRUCTURE^33^. This is consistent with conclusions of a recent study describing CGF40 as an alternative technique for source attribution in combination with comparative exposure assessment but not suitable using a source attribution model like the Asymmetric Island model^20^ since CGF40 do not provide enough details on genotypes compared with MLST^35^.

Based on large datasets of *C*. *jejuni* isolates from several putative sources of human contamination, the most likely origins of French campylobacteriosis from 2009 and 2015 were determined. In contrast to the majority of source attribution studies performed on MLST genes and using STRUCTURE software^20,22,37^, ruminants were the most common putative source of campylobacteriosis from 2009 in France (55%), and were equal to chicken in clinical cases from 2015 (38% for ruminant, 41–43% for chicken) based on MLST assignments. Nevertheless, this result is consistent with other source attribution studies^33,38^, and may support a greater role for the ruminant reservoir in campylobacteriosis^39^.

However, when host-segregating-based assignments were considered, as they showed a better accuracy in self-attribution than MLST, ruminant and chicken were equally important in France in 2009, but there were more attributions to chicken in 2015, comparable to other studies^19–21,31,34,40^. Despite a variation in the source attribution of clinical isolates from 2009 and 2015, both populations were mainly contaminated with agricultural *C*. *jejuni* which include isolates from chicken and ruminants. Contamination with chicken was especially associated with the consumption of broiler meat (undercooked)^1,19,34,41–44^. This is consistent with the high prevalence of *Campylobacter* spp. on carcasses and retail broiler meat in France estimated to 88% and 76% respectively^45,46^, and the important overlap between *C*. *jejuni* genotypes circulating in chicken and isolated in humans in France^29^.

Different risk factors were identified for human contamination by ruminants-associated *Campylobacter* spp. such as consumption of tripe or raw milk, barbecuing in non-urban areas, contact with garden soil or having a local and a regional tap water provider at home^19,34,37,43,44^. In addition to these, consumption of undercooked beef meat was identified as a risk factor for *C*. *jejuni* infections in France as well as in the Netherlands to a lesser extent^42,47^. However, despite a high prevalence of *Campylobacter* ssp. in French cattle^10^, the food-borne transmission of *Campylobacter* spp. is not clear, especially since no *Campylobacter* were detected in bovine meat in France^48^ in accordance with studies reporting rare beef or veal contamination^49–51^. On the other hand, cattle livers could be a non-negligible source of contamination in France since they constitute a popular dish in French cuisine and were shown to be highly contaminated by *Campylobacter* spp.^51,52^. As previously suggested^19,33,35^, contact with animals and the environmental contamination by ruminants, including water contamination, need also to be considered since *Campylobacter* spp. were shown to survive in bovine manure^53^ or during anaerobic digestion of livestock effluents in biogas plant^54,55^. Waterborne transmission of *Campylobacter* from ruminant to human has been previously reported^56^ and a recent 2-year study highlighted a high prevalence (80.7%) of *Campylobacter* spp. in environmental waters from intensive livestock farming areas in France^57^.

Implication of the environmental reservoir in our study, including environmental waters and wild birds, was low in 2009 (0–11% using MLST or the host-segregating markers) but slightly increased in 2015 (7–19% using MLST or the host-segregating markers). Our environmental-related estimates were in accordance with previous works^19,33,34,37,58^, which mainly associated these cases to consumption of untreated or private well water, practice of recreational activities related to water^59–61^, game consumption^34^, or contact with garden soil^19^, while consumption of drinking water in bottles were protective^60^. Consistent with this, contamination through the consumption of treated drinking water is unlikely in France as no *Campylobacter* were isolated from drinking water^16^, and from groundwater despite the detection in this case of *C*. *jejuni* and *C*. *coli* genomes in the samples^62^. However, it was reported that 50% of surface water upstream treatment plants were contaminated by *Campylobacter* spp. in Brittany, France^16^, suggesting that any failure in treatment (e.g. chlorination) could trigger to human contamination. This has been previously described worldwide^43,63^, as well as in France, where an agricultural contamination of groundwater was hypothesized^64^.

The role of wild birds in human contamination has been poorly investigated in France. As reported by Cody *et al*.^58^, several studies highlighted the contamination of equipment and surfaces in children playgrounds associated with a frequent hand-to-mouth behaviour in children^65^, and consumption of milk from bottle where the top had been pecked by birds^66^, as potential *Campylobacter* transmission routes from wild birds to human. In addition, isolation of *C*. *jejuni* belonging to wild bird-associated clonal complex (CC-177) in freshwater in France^67^ supports the previously described potential waterborne transmission of *Campylobacter* from wild birds to human^68^.

Companion animals including cat and dog were not highly involved in clinical cases in France (4% to 12% using MLST or the host-segregating markers), consistent with previous attributions^22,31^ and contrasting with the 25% of clinical cases attributed to pets in the Netherlands^32^. With regard to self-attribution tests, probabilities of correct assignment were generally low in pets regardless of the genotyping method used (MLST: 0.268 WGS: 0.272 CGF40: 0.0), suggesting an overlap of pets genotypes with those from the 3 others reservoirs. It is highlighted by the predominance of ST-45 in pets^32,69^, and its isolation in chicken^29,68,70^, environmental waters, wild birds^58,65,71^, and cattle^24,72^, indicating that chicken, ruminants or environment are likely to be significant sources of *Campylobacter* for pets through several transmission routes (e.g. food such as raw meat or offal). However, when pets are contaminated they may constitute a transmission route for chicken, ruminant or environmental-related *Campylobacter* to humans, suggesting that owning a companion animal increased human exposure to *Campylobacter* spp., emphasizing its role as risk factor^32,41,59^. Another potential scenario is the role of human in pets contamination, since these animals are likely to be fed with the same foods than their owner and especially with their food leftovers^32^.

Finally, our source attribution is not without limitations. While chicken and cattle *C*. *jejuni* collections show a national coverage^10,45,46^, pets and environmental populations may not be representative of *C*. *jejuni* from these reservoirs in France, as sampling surveys were locally conducted^57,67,73^. However, samplings were done on large period of time (6-month or 2-year period) to isolate a high number of strains in order to minimize this bias. In addition, the time span of strains isolation is important to consider, especially in a highly recombinant microorganism like *Campylobacter*, in which MLST genotypes were shown to be increasingly different over time^74^. However this bias can be nuanced as several studies identified a temporal stability in the population structure of isolates from chicken, wild birds and clinical cases^75–77^. Moreover, clinical cases studied here may not be representative of all notified French campylobacteriosis *C*. *jejuni* cases, since surveillance of campylobacteriosis in France is not mandatory leading to underestimate its incidence^78^. Therefore, it was not possible to get a representative collection of all cases occurring in France. However, in  our study, we selected *C*. *jejuni* campylobacteriosis cases from the 10 most populated departments which represent 26% of the French population with 17,585,983 inhabitants (official statistics in 2014 from the National Institute of Statistics and Economic Studies). Lastly, the comparison of our results with studies using different source attribution models could be discussed, nevertheless, for the two main models used for source attribution (STRUCTURE and Asymetric island model), Sheppard *et al*.^20^ showed that they produced consistent results.

In conclusion, a variation was observed in assignments of French clinical cases between 2009 and 2015 and according to the genotyping method used. The host segregating markers were the most accurate in self-attribution especially for chicken isolates and apart from environmental isolates. A predominant role of agricultural reservoirs (chicken and ruminant) was observed in campylobacteriosis from 2009 and 2015 in France, emphasizing the importance of intervention strategies to control *Campylobacte*r in hosts in order to decrease the human burden. It is especially true for cattle where the environmental contamination by *Campylobacter* of human might be more important than the foodborne pathway, addressing the question of transmission routes for *Campylobacter* from ruminant to human, as no clear evidence is available. Nevertheless, since host-segregating markers allowed a higher accuracy in assignments of chicken isolates than MLST loci, it suggests that the importance of chicken in campylobacteriosis may be underestimated using MLST and could be more important than currently described. Finally, combining molecular and epidemiological approaches of source attribution may be of interest for further investigation of possible transmission routes for *Campylobacter*. In relation with French consumption habits and behaviour, this combining approach would be helpful to better understand campylobacteriosis epidemiology in France, and how different trends of source attribution can be obtained compared with our neighbouring countries.

## Material and Methods

### Clinical, animal, and environmental isolates

A total of 2132 *C*. *jejuni* isolates were collected between 2008 and 2016 in France, and characterized in this, and previous studies^10,24,29,77,79^. Clinical isolates were obtained from the National Reference Centre for *Campylobacter* and *Helicobacter* in France. In 2009, 3754 isolates from clinical cases of campylobacteriosis were obtained from 348 diagnostic bacteriology laboratories in public hospitals and private laboratories belonging to the National Surveillance System of campylobacteriosis in France^80^. *C*. *jejuni* was the most common species representing 81.4% (n = 3054) of *Campylobacter* spp. isolates^80^. Of these, *C*. *jejuni* isolates from the 10 most populated regions in France (n = 143) were considered for genotyping (CGF40, MLST, WGS) and included in this study^24,29^. In 2015, 5722 *Campylobacter* spp. isolates from campylobacteriosis were reported by laboratories from the National Surveillance System in France and identified by the National Reference Centre^81^. Of the 4704 *C*. *jejuni* isolates^81^, 371 isolates obtained from the 10 departments selected in 2009, were considered in this study. All isolates were successfully characterized using CGF40^77^, and a subset (n = 79) was characterized using MLST and WGS.

In addition, isolates originating from 4 potential sources of human infection were included in this study: (i) chickens, 644 isolates from 2008 and 2009, representative of the broiler production chain in France (national coverage of 9-month and 12-month sampling surveys performed at retail and slaughterhouse levels respectively)^7,46^; (ii) cattle, 42 isolates from 2013, and 649 from 2016 representative of the French cattle production (6-month sampling survey at slaughterhouse level allowing the analyses of 959 samples from 282 farms distributed among 32 French departments representative of the French production of cattle)^10^; (iii) environment, 122 isolates from 2013 to 2015 and from freshwater, sea water, sediment or mussels; (iv) pets, including 161 cat and dog isolates from 2014 and 2015. Isolates details and source publications are detailed in supplementary Table S1.

### DNA extraction

Isolates stored at −80 °C, were subcultured onto *Campylobacter* selective blood-free agar (Karmali, Oxoid) in microaerobic conditions (85% N_2_, 10% CO_2_, 5% O_2_) at 42 °C for 48 h. Genomic DNA was extracted from one-day single-colony cultures incubated at 37 °C using the kit QiaAMP DNA Mini Kit (QIAGEN) and quantified using the Qubit® 2.0 fluorometer and the Qubit dsDNA HS Assay kit (Invitrogen) following manufacturers’ recommendations.

### Comparative Genomic Fingerprinting (CGF40)

CGF40 fingerprints were generated from 8 Multiplex PCRs according to primer sets previously published^25^ as well as experimental conditions^29^. The PCR results were converted into binary data corresponding to the absence (0) or the presence (1) of each of the 40 markers in the bacterial genomes and the CGF40 fingerprints of these 40 genes (CGF40) were stored into BioNumerics® software (v 7.6, Applied Maths, Belgium). Each binary CGF40 fingerprint was then used in the source attribution model to assign an origin to clinical *C*. *jejuni* isolates from 2009 and 2015. To perform this, all clinical, animal and environmental isolates were previously genotyped using CGF40^10,29,77,79^.

### MultiLocus Sequence Typing (MLST)

**Figure 4:**
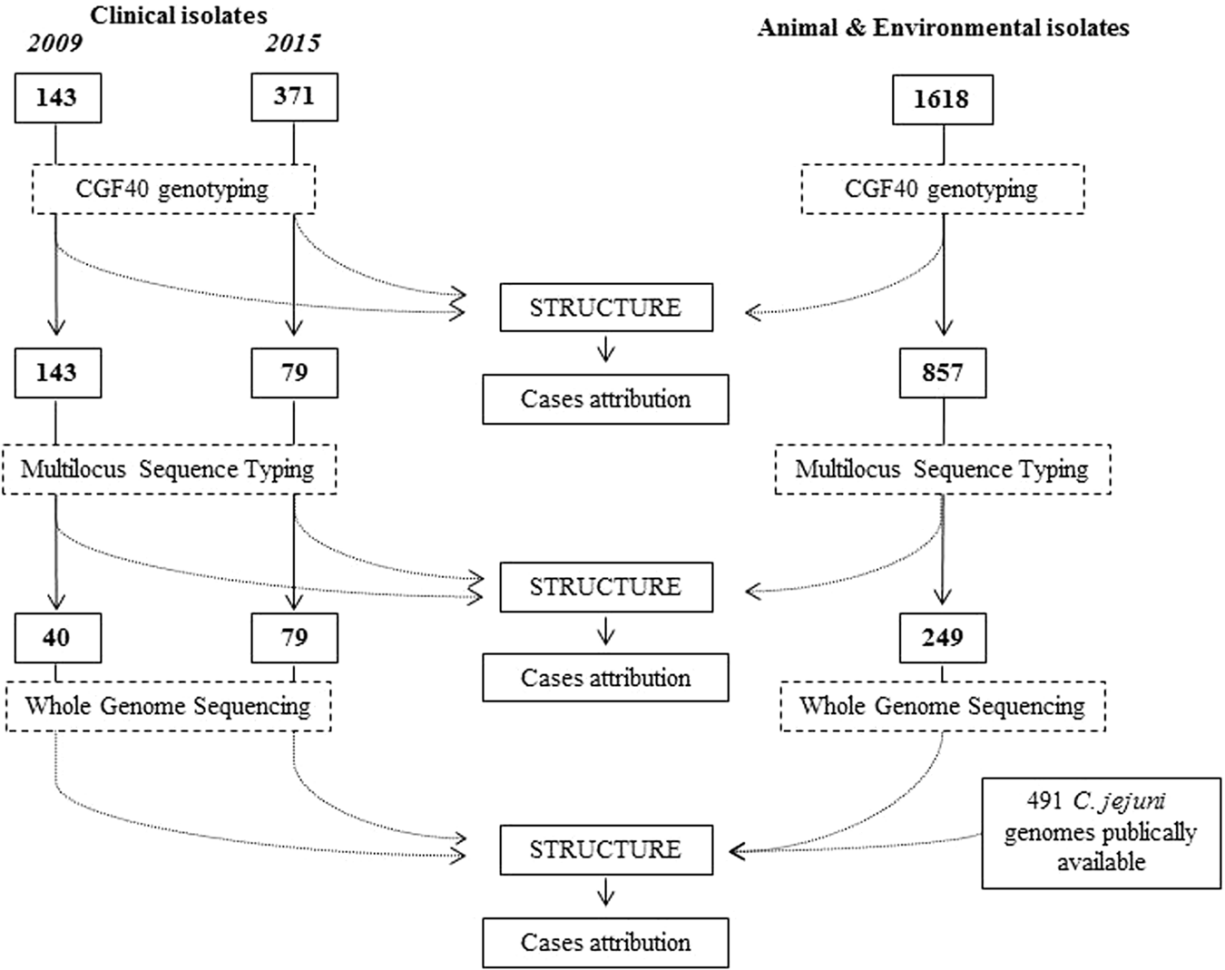
Experimental design of the study.

Alleles of the seven housekeeping MLST genes (*aspA*, *glnA*, *gltA*, *glyA*, *pgm*, *tkt* and *uncA*) were determined as previously described^29^. Alleles, sequence types (ST) and clonal complexes (CC) of isolates from cattle, pets, environment and clinical cases from 2015 were determined from whole genome sequence (WGS) data and by comparison of the sequences to the PubMLST database (http://pubmlst.org/campylobacter) on BIGSdb^82^. MLST characterization through WGS was performed using a subset of isolates from cattle, pets, environment and clinical cases from 2015, keeping the same proportion as for CGF40 genotypes. The global experimental design is presented in Fig. 4.

### Whole Genome Sequencing (WGS)

Genomes were sequenced using the Ion Torrent technology on an Ion Torrent Proton machine (Life Technologies) according to previously published conditions^24^. Assemblies were produced by either MIRA version 4.0rc1^83^ or SPAdes 3.1.1^84^. Among 156 *C*. *jejuni* genomes newly sequenced, an average of 150 contigs was obtained with a median value of 62 contigs. The average of the total assembled sequence length is 1,715,087 bp (Supplementary Table S2).

French genomes sequenced in this study or previously^24^ were augmented with 491 genomes of *C*. *jejuni* isolated from chicken, ruminant, environmental water and wild birds from different countries and published in previous studies (Fig. 4)^85–88^. This gave a total of 859 *C*. *jejuni* genomes to constitute our study dataset (Supplementary Table S1) in which allelic variations among 15 host segregating markers^24^ was assessed for the source attribution study. These host-segregating markers were preferred to whole genome to perform source attribution, as their potential in source attribution has been demonstrated^24^, while the whole genome was shown to not improve assignment compared with MLST^23^.

### Accuracy of the several genotypes data in source attribution through self-attribution tests

To assess the accuracy of attribution probabilities obtained with each genotyping method, self-attribution tests were performed within the different host populations, as described in previous studies using MLST or the host-segregating markers^20,24^. Random subsets of twenty isolates from each hosts population were assigned to a dataset from unknown origin and 10 independent self-attribution tests were performed to assign these isolates to a source. Accuracy of genotyping methods were considered as significantly different when no overlap of their 95% confidence interval were observed. Experimental conditions used were identical to those used to attribute a source to the clinical isolates.

### Molecular source attribution of the clinical isolates

Probabilistic assignment of French human isolates from 2009 and 2015 to their most likely origin was performed separately using STRUCTURE software^89^. This software estimates the most likely origin of clinical isolates according to the similarity in alleles frequencies among the potential host populations and by assuming that each host population is characterized by its own set of allelic frequencies. CGF40 fingerprints, MLST profiles, and allelic profiles of the 15 host-segregating loci^24^ were used to attribute a source to clinical isolates according to previously published conditions^24^. Briefly, 100,000 burn-in steps with 100,000 subsequent iterations were run in STRUCTURE using the no-admixture model, assuming uncorrelated gene frequencies and using the STARTATPOPINFO parameter turned on. Clinical isolates were distinguished from host populations isolates using POPFLAG.

Host datasets used as a reference to probabilistically attribute a source to clinical isolates included isolates from 3 putative sources of contamination (chicken, cattle and environment) (Supplementary Table S1). Pets were added as a source of infection in a second analysis since their role in campylobacteriosis as reservoir or vector is not fully elucidated^32,90,91^.

### Accession number(s)

Genome sequences generated as part of this study belong to the BioProject PRJNA357677 and were deposited in SRA (SRR6914212 to SRR6914375; see supplementary Table S2). The assemblies of genomes sequenced in earlier studies can be found in Dryad (https://doi.org/10.5061/dryad.28n35 and https://doi.org/10.5061/dryad.m86k3) and NCBI (BioProject PRJNA312235 and BioProject PRJNA357677).

## Electronic supplementary material

Supplementary material
